# Interplay of Ribosomal DNA *Loci* in Nucleolar Dominance: Dominant NORs Are Up-Regulated by Chromatin Dynamics in the Wheat-Rye System

**DOI:** 10.1371/journal.pone.0003824

**Published:** 2008-12-02

**Authors:** Manuela Silva, H. Sofia Pereira, Miguel Bento, Ana Paula Santos, Peter Shaw, Margarida Delgado, Nuno Neves, Wanda Viegas

**Affiliations:** 1 Centro de Botânica Aplicada à Agricultura, Instituto Superior de Agronomia, Technical University of Lisbon, Tapada da Ajuda, Lisboa, Portugal; 2 Cell and Developmental Biology, John Innes Centre, Colney, Norwich, United Kingdom; 3 Universidade Lusófona de Humanidades e Tecnologias, Lisboa, Portugal; 4 Departamento de Ciências da Vida, Faculdade de Ciências e Tecnologia, Universidade Nova de Lisboa, Monte da Caparica, Caparica, Portugal; National Institute on Aging, United States of America

## Abstract

**Background:**

Chromatin organizational and topological plasticity, and its functions in gene expression regulation, have been strongly revealed by the analysis of nucleolar dominance in hybrids and polyploids where one parental set of ribosomal RNA (rDNA) genes that are clustered in nucleolar organizing regions (NORs), is rendered silent by epigenetic pathways and heterochromatization. However, information on the behaviour of dominant NORs is very sparse and needed for an integrative knowledge of differential gene transcription levels and chromatin specific domain interactions.

**Methodology/Principal Findings:**

Using molecular and cytological approaches in a wheat-rye addition line (wheat genome plus the rye nucleolar chromosome pair 1R), we investigated transcriptional activity and chromatin topology of the wheat dominant NORs in a nucleolar dominance situation. Herein we report dominant NORs up-regulation in the addition line through quantitative real-time PCR and silver-staining technique. Accompanying this modification in wheat rDNA trascription level, we also disclose that perinucleolar knobs of ribosomal chromatin are almost transcriptionally silent due to the residual detection of BrUTP incorporation in these domains, contrary to the marked labelling of intranucleolar condensed rDNA. Further, by comparative confocal analysis of nuclei probed to wheat and rye NORs, we found that in the wheat-rye addition line there is a significant decrease in the number of wheat-origin perinucleolar rDNA knobs, corresponding to a diminution of the rDNA heterochromatic fraction of the dominant (wheat) NORs.

**Conclusions/Significance:**

We demonstrate that inter-specific interactions leading to wheat-origin NOR dominance results not only on the silencing of rye origin NOR *loci*, but dominant NORs are also modified in their transcriptional activity and interphase organization. The results show a cross-talk between wheat and rye NORs, mediated by ribosomal chromatin dynamics, revealing a conceptual shift from differential amphiplasty to ‘mutual amphiplasty’ in the nucleolar dominance process.

## Introduction

Chromatin, the living form of genetic information in eukaryotes, has particular organization and distribution patterns in the nucleus that are related to gene expression as observed in several biological systems, ranging from animals to plants. One of the most widespread features of this relationship concerns the association of decondensed chromatin (euchromatin) with transcriptional activity, based on a greater physical availability of DNA sequences to the transcription machinery. Heterochromatin, which is the cytological representation of chromosome domains that undergo dense packaging are mainly composed of repetitive DNA sequences [1], [2] (review in [3]), and is involved in transcriptional silencing of genes located in cis or in trans co-arrangements by spreading of heterochromatinization [4]. Additional significance of heterochromatin also relies on its involvement in the RNA interference pathways that lead to transcriptional and post-transcriptional gene silencing [5]. Chromosome domains and their topology, in addition to other functionally relevant nuclear landscapes (e.g, transcription *foci*, RNA processing, DNA repair), point to the importance of functional compartmentalization of the nucleus [6]. One of the most representative features of intranuclear compartments ascribed to particular nuclear functions is the nucleolus, which largely results from transcription of 45S ribosomal RNA (rRNA) genes [7]–[9]. Besides nucleolar involvement in several cellular processes (review in [10]), the nucleolus has, nevertheless, a universal and fundamental function as the ribosome sub-unit production centre. The formation of the nucleolus is primarily dependent on the transcriptional activity of competent NORs (nucleolus organizer regions) that are composed of hundreds to thousands tandem copies of rRNA genes [9], [11]. It has also been established that in virtually all eukaryotes there is an excess of cellular rRNA genes in relation to ribosome needs for protein synthesis [8], [12]. Hence, most of the NORs have only part of their rRNA gene arrays being transcribed at any particular time, while the remaining arrays adopt a heterochromatic configuration forming knobs at perinucleolar location [9], [11]. In addition, rDNA physical organization seems to be correlated with the dynamic topology of rDNA *loci*. In fact, elegant studies in humans showed that NORs become associated in one large perinucleolar knob at early G1 phase [10], [13], a tendency also detected in *Arabidopsis thaliana* through the observation of frequent association between NOR-bearing chromosomes [14].

In *A. thaliana* the regulation of rRNA gene array availability for transcription has shown to be mechanistically linked to epigenetic modulation in nucleolar dominance phenomena where whole-NOR epigenetic silencing is commonly observed in hybrids and polyploids (e.g. *A. suecica*, [11], [15]). This process occurs in Drosophila [16], Brassica [17] and triticale [18] when only NORs from one progenitor are transcriptionally active and contribute to nucleolus formation dominating over the rRNA genes of the other species that are rendered silent. The cytological event was first described in Crepis hybrids, when Navashin pointed out morphological changes on a group of chromosomes of one progenitor, detecting the disappearance of their secondary constrictions where rRNA gene arrays are located [11], [12], [19], [20]. At that time the process observed by Navashin was termed differential amphiplasty, since only one parental genome in several Crepis hybrids suffers consistently the modifications on chromatin morphology - the absence of secondary constrictions, i.e. NOR chromatin full compaction. Considering that studies on nucleolar dominance were focused in silenced NORs behaviour [11], [12], information on the transcriptional level and chromatin organization of dominant NORs is still sparse and needed for the growing understanding of the dynamic behaviour of these key genomic domains. To address this topic, the wheat-rye system was used in the current investigation. This biological system shows nucleolar dominance of wheat (*Triticum aestivum* L.)-origin NORs over rye (*Secale cereale* L.)-origin NOR *loci* [18], which is mediated by epigenetic cues such as DNA methylation [21], [22]. Regarding the organization of ribosomal chromatin, rye NORs show a heterochromatic centromere proximal domain from which decondensed rDNA portions emerge toward the nucleolus [23], [24]. In wheat the pair of major NORs on chromosome 1B present an organization similar to that of rye NORs, whereas the other pair of major NORs on chromosome 6B presents two heterochromatic domains (a centromere proximal and a distal one), with the decondensed rDNA domain positioned between them [25], [26]. Using this system we analyzed the potential changes in wheat-origin rDNA transcriptional activity, as well as in their organization and topology. We report that nucleolar dominance is a process where NORs of both parental species are modified in the wheat-rye combination (although in opposite functional directions), in contrary to the differential amphiplasty concept as described by Navashin in 1930s [19] affecting only under-dominant NORs. Our model points out that dominant NORs are up-regulated by rDNA chromatin co-dynamics with under-dominant rDNA *loci*, and presumably mediated by epigenetic modulation.

## Results and Discussion

### Expression of wheat rDNA *loci* is enhanced by rye nucleolar chromosomes

**Figure 1:**
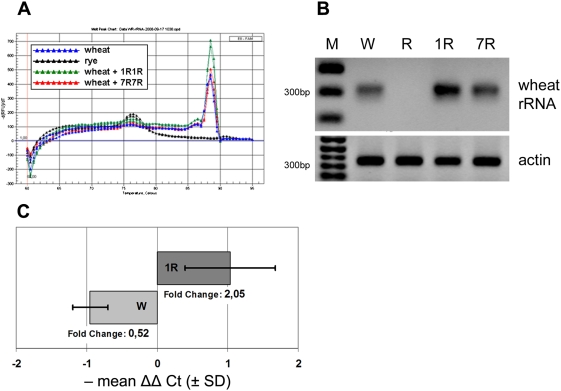
Quantitative real-time PCR of wheat rRNA in wheat and wheat addition lines. A The melt curves for two replicates of cDNA isolated from wheat (blue), rye (black), wheat+1R1R (green), and wheat+7R7R (red) amplified with primers specific for wheat rRNA are shown. A single melt peak with Tm = 88.5 in the three genomes containing wheat genetic material indicate a single amplification product. Due to the specificity of primers for wheat rRNA, there is no amplification of rye rRNA. B Quantitative real-time PCR products separated by gel electrophoresis. The results indicate greater expression of wheat-specific rRNA in the addition line wheat+1R1R (1R) in comparison to wheat (W) and addition line wheat+7R7R (7R). No amplification product was observed in rye (R). Actin controls are shown and M is the molecular weight marker (1 kb^+^, 300 basepair band is shown on the left). C Transcription levels of wheat rRNAs in wheat and wheat+1R1R in respect to wheat+7R7R. Quantitative real-time PCR threshold cycles (Ct) were equilibrated with actin for wheat, wheat+1R1R, and wheat+7R7R (delta Ct). Wheat+7R7R mean delta Ct was utilized to calculate mean delta delta Ct (mean ΔΔ Ct) of two replicates of two wheat and wheat+1R1R cDNA dilutions. The graph illustrates – mean ΔΔ Ct ± standard deviation (SD) for wheat and wheat+1R1R, and the associated fold changes in transcription (2 ^−mean ΔΔ Ct^).

Quantitative real-time PCR was used to evaluate wheat-specific rDNA transcription levels in wheat and wheat+1R1R addition line. The actin control amplified similar quantities of a single product with approximately 350 bp and a melting temperature (Tm) of 86°C in all four genomes analyzed (Figure 1B). However, there were marked differences in the levels of wheat-specific rRNA expression between wheat, rye and the addition line wheat+1R1R (Figure 1A and B). Importantly, the wheat-specific primers amplified a single cDNA product with the expected size of approximately 300 base pairs (Supporting Information, Fig S1) and with a T_m_ = 88.5°C in the three genomes containing wheat genetic material, but no amplification product was observed in rye. Mean delta delta C_t_ ± standard deviation of wheat-specific rRNA was calculated for wheat and the wheat+1R1R addition line utilizing the addition line wheat+7R7R as standard. This resulted ΔΔC_t_ = 0.95±0.46 for wheat, and ΔΔC_t_ = −1.03±0.65 for the addition line wheat+1R1R (graphic representation in Figure 1C). These results indicate significantly different expression levels of wheat-specific rRNA between wheat and wheat+1R1R (Student's *t* test, p = 0.0025). In comparison to the wheat+7R7R addition line, the fold variation in the expression of wheat-specific rRNA was calculated to be 0.52 (approximately 50% less) in wheat and 2.05 (approximately 200% greater) in wheat+1R1R. In conclusion, the expression of wheat-specific rRNA is approximately four fold higher in the wheat+1R1R addition line in comparison to wheat.

**Figure 2:**
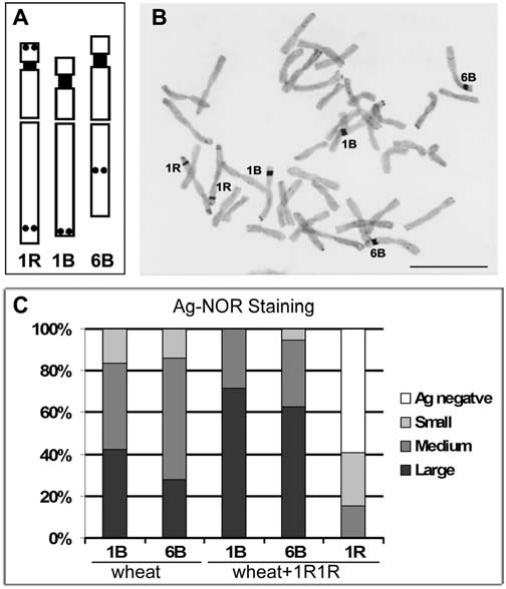
Evaluation of NOR activity in wheat and wheat-rye addition line (wheat+1R1R). Silver-staining technique in root-tip metaphase cells. A shows the schematic representation of the NOR bearing chromosomes (NORs black square) and the diagnostic silver bands (black circles) allowing the identification of nucleolar chromosomes 1B, 6B, and 1R. B shows a root-tip metaphase cell of the wheat-rye addition line (wheat+1R1R), with six Ag-NORs. In this plate the 1B and 6B NORs are classified as large while 1R NORs are classified as medium. Bar = 10 µm. C represents the graphic for the frequency of different classes of Ag-NORs for each rDNA locus in root-tip metaphase cells of wheat and wheat+1R1R. Values result from the analysis of 50 c-metaphase cells in each genotype, and the differential distribution for 1B and 6B NORs between the two lines was confirmed by Chi-squared test (p<0.001).

In order to cytologically analyze rDNA expression levels of the 1B, 6B and 1R NORs a silver-staining method was used. The intensity and size of silver-positive metaphase NORs was recently proved to be positively correlated with transcriptional activity (determined by S1 nuclease protection assays) in rye [27]. Furthermore a characteristic banding pattern, resulting from an improved silver-staining procedure [23], allowed for the precise identification of wheat and rye NOR-bearing chromosomes (Figure 2A). The evaluation of NOR activity was analyzed per nucleolar chromosome, through a comparison between Ag-NOR dimension and its respective satellite length as a standard unit (Figure 2B and C; see Materials and Methods). Ag-NORs frequencies with different dimensions are presented in Figure 2C. In the wheat euploid line both 1B and 6B NORs were silver-positive in virtually all cells, indicating that these rDNA *loci* can be considered as constitutively active. Most metaphase cells show large to medium 1B Ag-NORs, whereas the 6B NORs are usually of medium sized, corroborating the expression hierarchy of major NORs in wheat: 1B>6B [22], [28].

In the wheat+1R1R line (Figure 2B and C), silver-staining revealed 40% of cells with Ag-stained NORs of rye origin, and the majority were only faintly labelled small NORs. These results confirmed a clear effect of nucleolar dominance of wheat over rye, since in a rye environment 1R NORs show strong labelling, mostly belonging to large NOR class in all nuclei [23]. On the other hand, the frequency of differently sized Ag-NORs of wheat-origin was significantly different between the two genotypes analysed (Chi-square test, p<0.01). An evident increase in the frequency of large wheat-origin Ag-NORs was detected in wheat+1R1R when compared with the wheat, 30% more of large 1B and 35% more of large 6B Ag-NORs (Figure 2C) revealing the modulation of dominante NORs expression patterns in nucleolar dominance situations. Further, the presence of rye nucleolar chromosomes did not affect the intrinsic mechanisms associated with the differential expression of the wheat NORs from its B ancestral parental genome, since there is no significant difference between the relative expression patterns between 1B and 6B NORs in wheat and in wheat+1R1R. The comparison made indicated that the addition of rye nucleolar chromosomes to the wheat genome, besides leading to the silencing of rye origin rDNA *loci*, induced an overall enhancement of expression in wheat-origin rDNA *loci*, i.e. of the dominant rRNA gene arrays. The up-regulation of dominant NORs in a situation of nucleolar dominance has only been referred in Brassica hybrids following treatment with the hypomethylating drug 5-aza-deoxicitidine [29]. In natural conditions, a cross-talk between dominant and underdominant NORs is now disclosed.

### rRNA gene transcriptional activity is related to ribosomal chromatin topology in wheat

**Figure 3:**
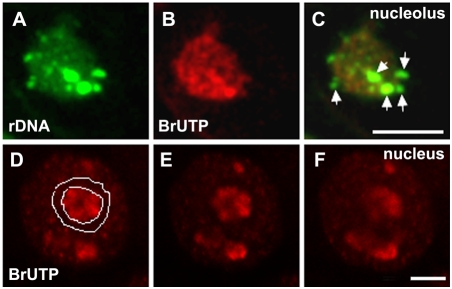
Ribosomal chromatin organization and transcription sites in wheat. A–C Nucleolar labelling of rDNA chromatin (A, green) and of transcription sites detected by BrUTP incorporation (B, red) in root sections of wheat. A and B are projections of single confocal sections spanning the nucleolus; C is the merging of rDNA and BrUTP images. No transcription *foci* are detected in perinucleolar knobs of ribosomal chromatin (C, arrows) whereas intranucleolar rDNA is associated with BrUTP revealing transcriptional activity (orange labelling resulting from overlapping of green and red channels). D–F Nuclear transcriptional labelling detected by BrUTP incorporation. D, E and F are individual consecutive confocal sections of the nucleus. Transcription sites are overall dispersed throughout the nucleus and in the nucleolar compartment. Note the absence of transcription label in the chromatin encircling the nucleolus (indicated by the lines in D). Bar = 5 µm.

Confocal microscopy analysis of wheat interphase nuclei after FISH probing to the rRNA genes allowed for the identification of condensed ribosomal chromatin, with larger knobs being allocated at the nucleolar periphery and smaller dots with an intranucleolar positioning (Figure 3 A–C). Therefore, interphase nuclei from wheat meristematic root cells exhibited two distinct fractions of compact ribosomal chromatin, concerning their position in relation to nucleolar mass. These organizational features of wheat rDNA at interphase have been previously suggested [24], [26] as being transcriptionally significant. In order to ascribe the true nature of this relation, nuclei were simultaneously labelled for transcription *foci* (BrUTP incorporation) and probed for rDNA chromatin (Figure 3 A–C). Both in wheat and in the wheat-rye addition lines, nuclei showed an almost homogenous distribution of small BrUTP *foci*, compatible with the occurrence of transcription throughout wheat nuclei [30]. An intense and concentrated labelling was also detected inside the nucleolus, comprising many closely packed transcription *foci* showing a stronger transcriptional activity in this nuclear compartment, as shown for human cells [31]. In contrast, only a residual presence of BrUTP incorporation was observed in the chromatin domains surrounding the nucleolus (Figure 3 D–F). This fact show the almost total transcriptional silencing of these chromatin regions, parallel to the observations made in mouse neurons, where nucleoli seem to be encaged by perinucleolar heterochromatin domains consisting of several classes of inactive DNA [32]. Other studies have also reported that transcriptionally inactive DNA, including the inactive X-chromosome in mammals, is recruited to nucleolar surroundings (reviews in [7], [33]). Our findings in plant cells indicate that this kind of nuclear chromatin organization may reflect a widespread feature of high eukaryotes.

Regarding the compact rDNA fractions described above, the condensed ribosomal chromatin regions inside the nucleolus (rDNA dots) show colocalization with BrUTP incorporation, whereas the domains of condensed rDNA chromatin localized adjacent to the nucleolar boundaries (rDNA knobs) do only contain residual transcriptional labelling (Figure 3). These results are in accordance with our previous findings in the allopolyploid *A. suecica* [15]: silent NORs (derived from the *A. thaliana* progenitor) are present as rDNA perinucleolar knobs and positive through immunodetection for particular epigenetic marks linked with transcriptional silencing. This useful approach can not be followed in the wheat system, since species with large genomes (like wheat and its relatives) have no specific distribution of epigenetic tags in discrete domains [34]. However, there is strong evidence that epigenetic pathways (namely DNA methylation) mediate rRNA gene transcription in cereals [21], [22] may be involved in the establishment and maintenance of particular states of ribosomal chromatin in the wheat-rye system. Moreover, artificially induced DNA hypomethylation has already proved to alter rDNA conformation in wheat [35] and rye [27].

### Large-scale organization of wheat ribosomal chromatin is modified by rye nucleolar chromosomes

**Figure 4:**
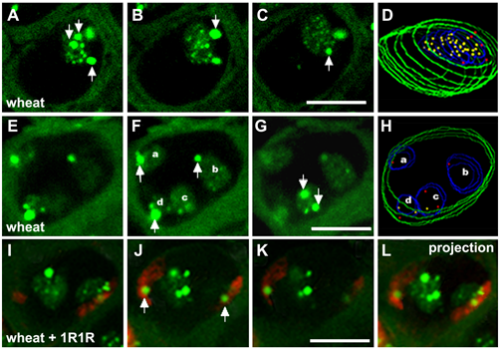
Ribosomal chromatin organization in wheat and wheat-rye addition line (wheat+1R1R) nuclei. In situ hybridization to rDNA in root sections of wheat and wheat-rye addition line (wheat+1R1R). A–C and E–G are wheat single consecutive confocal sections of interphase nuclei with one or four nucleoli respectively (perinucleolar knobs are indicated with arrows). D and H are models from the complete 3D data stack shown in A–C and E–G, respectively, where intranucleolar dots are represented in yellow and perinucleolar knobs in red. The single nucleolus of the nucleus shown in A–D has five perinucleolar knobs (arrows in A, B and C). The nucleus shown in E–H has four nucleoli, where one perinucleolar knob is visible in nucleoli marked as a, b and d (arrows in F), while the fourth nucleolus (c) shows two perinucleolar knobs (arrows in G). I–L shows a wheat-rye (wheat+1R1R) interphase nucleus probed for the rDNA (green) and for rye chromosomes 1R (red). I–K are single confocal consecutive sections and L corresponds to the projection of the 3D data stack, showing the presence of two nucleoli. In these plates it is possible to distinguish the wheat and rye rDNA perinucleolar knobs, since the later overlap with 1R chromosomes (white arrows in J). Bar = 10 µm.

Earlier observations in wheat found four nucleoli when interphase resumes, resulting from the activity of the two already mentioned pairs of wheat major NORs, present at chromosomes 1B and 6B [28]. Considering the size of the ribosomal chromatin condensed domains described above for wheat interphase nuclei, perinucleolar chromatin knobs of rRNA gene arrays have a diameter varying between 3 to 4 µm, and intranucleolar condensed dots ranged among 1–2 µm in diameter (Figure 4A). In addition, the 3-D analysis allowed the identification of number variation involving both types of rDNA condensations - intranucleolar dots and perinucleolar knobs. Intranucleolar rDNA condensations are almost absent in nuclei with four nucleoli; however they are abundant in nuclei with one nucleolus. The data suggests that the number of intranucleolar dots (and hence the organization of ribosomal chromatin inside nucleoli) is dependent upon nucleolar fusion. Regarding the rDNA condensed domains at the nucleolus periphery, the average of perinucleolar knobs per nucleolous varies from 5.4 (±0.8) in nuclei with one nucleolus (n = 70 nuclei) and 1.4 (±0.6) in nuclei with four nucleoli (n = 28 nuclei), which corresponds to the same value per nucleus. In contrast to the observation made for intranucleolar dots, these results indicate that nucleolar fusion does not alter the number of perinucleolar knobs per nucleus. In addition, the mean value of 1.4 perinucleolar knobs per nucleolus observed in wheat nuclei with four nulceoli is fully justified taking into account the specific interphase organization patterns of 1B and 6B NORs: the 1B NOR exhibit one perinucleolar knob proximal from the centromere, while the 6B NOR organizes two condensed rDNA blocks at the nucleolar boundary (one centromere-proximal and another centromere-distal) resulting from decondensation and transcription of intercalary 6B rRNA gene arrays [25], [26].

This observed invariability for the number of rDNA perinucleolar blocks in wheat nuclei is not however detected in other biological systems. In fact, Hernandez-Verdun and collaborators have shown that in HeLa cells at the exit of mitosis, nucleolar biogenesis is tightly coupled with movements of different NORs with respect to one another, where the distinct NORs assemble in a single domain and contribute all to a single nucleolus at early G1 [13]. Our observations in wheat mean that each NOR and respective nucleolar chromosome maintains its identity throughout the cell cycle as distinguishable domains regardless of the number of nucleoli. Interestingly this difference in rDNA topology observed in wheat and human nuclei is accompanied by a global distinctive characteristic concerning chromatin topology in the two systems: while the large wheat genome is organized in a Rabl configuration, consisting in telomere and centromere clustering at opposite poles of the nucleus [30], [35]. The human genome is not organized in such a orderly fashion as Rabl configuration, but instead the centromeres are distributed at nuclear or nucleolar periphery [36], [37]. In *A. thaliana*, a plant species with small genome and without Rabl chromatin disposition, rDNA chromatin organization studies show also a preferential proximity and even association between nucleolar chromosomes [14]. Considering this comparative information we suggest that rDNA topology patterns and nucleolar biogenesis may be related to the large-scale genomic organization at interphase.

In the wheat-rye addition line wheat+1R1R, where the pair of rye nucleolar chromosomes 1R is introgressed in the wheat genome, FISH with rye genomic DNA shows rye chromosomes 1R stretching across the nucleus, with the chromosome arms closely associated (Figure 4 I–L), in agreement with the Rabl model of interphase organization in cereals with large genomes [30]. In the wheat-rye addition line the rDNA chromatin also shows perinucleolar condensed knobs and intranucleolar condensed dots at interphase. The occurrence of small condensed blocks inside the nucleolus showed to be also dependent on nucleolar fusion, as observed in the wheat euploid line. However, regarding the number of large perinucleolar knobs of ribosomal chromatin, the addition line wheat+1R1R exhibits a significant difference in comparison to euploid wheat. In nuclei of this line probed simultaneously for rye genomic DNA and ribosomal sequences, rye-origin NORs can be easily detected (by the overlapping signals of both probes), hence discriminating the genomic origin of the NORs. This approach detected rye-origin NORs as condensed knobs proximal to the nucleoli, suggesting their low transcriptional activity, in accord with the silencing of rye origin rRNA gene arrays in the presence of wheat genome (i.e. nucleolar dominance, [21]). Of the large knobs of rRNA gene arrays, two were identified as belonging to 1R NORs in all the nuclei analyzed; the remaining knobs are from wheat major NORs 1B and 6B and always associated with nucleoli. The analysis of rDNA knobs from wheat revealed a significant decrease (Student's *t* test, p<0.0001) in the mean number of perinucleolar knobs per nucleolus (3.9±0.6 in wheat+1R1R, n = 74 nuclei), in comparison to the values obtained in wheat nuclei (5.4±0.8). Furthermore, the size of the observed condensed wheat rDNA blocks was similar in both lines (3 to 4 µm) suggesting that the reduction in number of perinucleolar knobs in the addition line did not result from their association at perinucleolar position but from a decrease in the rDNA condensed fraction. The same technical approach was followed in the wheat-rye addition lines wheat+6R6R and wheat+7R7R (rye chromosomes 6 and 7 do not contain rRNA genes), and no modification in wheat-origin ribosomal chromatin organization and topology was detected, with similar results to those observed in the euploid wheat (data not shown). Our proposal is that the presence of rye-origin NORs in a wheat background is responsible for the preferential decondensation of rDNA from wheat-origin, leading to the disappearance on average of 1 to 2 wheat perinucleolar rDNA blocks in the wheat-rye addition line.

### Concluding remarks

From our results, we propose that the presence of wheat and rye ribosomal chromatin in the same nucleus induces a mutual amphiplastic process instead of a unidirectional one, since the reduction of the activity of under-dominant NORs from rye origin [21] was associated with an increase of the activity of wheat-origin dominant rRNA gene arrays (this work) in comparison with their original genomic backgrounds. In wheat and rye, DNA methylation has been previously associated with rRNA gene expression [22], [27]. The silencing process of rye origin NORs in the wheat-rye system was also shown to be dependent of cytosine methylation status within rye rDNA intergenic spacer [21]. Therefore we expect that the bi-univocal interaction between NOR *loci* is associated with epigenetic modifications in *trans* that affect both the under-dominant (i.e. silent) NORs and the dominant rRNA gene arrays. This enlightens the significance of genome responses to challenges forwarded by Barbara McClintock in the mid 1980s [38]. This model is also in agreement with results that show that the epigenetic modifications that affect ribosomal *loci* expression are targeted to NORs rather than to particular rRNA genes [39]. Our model for rDNA transcription and organization seems also to associate with overall nuclear architecture of chromatin, namely Rabl versus non-Rabl interphase configurations. More studies on ribosomal chromatin topology and nucleolus biogenesis in other systems will contribute determinately for the understanding of the functional complexity of genome organization.

## Materials and Methods

### Plant material

Seeds from hexaploid wheat (*Triticum aestivum*, 2n = 6x = 42, genome designation AABBDD) cv. Chinese Spring, and from wheat-rye addition lines wheat+1R1R, wheat+6R6R and wheat+7R7R (wheat cv. Chinese Spring plus rye chromosome pairs 1, 6 and 7, 2n = 44), were germinated for 3 days at 25°C and further grown at climatic chambers with a photoperiod of 16 hours light (20°C)/8 hours dark (20°C) cycle. Root tips from 3 days old seedlings were used for cytological analysis and leaves from three week old plants were used for RNA extraction. Seeds stocks from all the genotypes were originally obtained from the USDA–Sears collection, Columbia, Mo and were stored at the Secção de Genética, Instituto Superior de Agronomia, Lisbon.

### Evaluation of rDNA expression levels through quantitative real-time PCR

Wheat-specific rDNA transcription levels were analyzed by Quantitative real-time-PCR with the BIO-RAD IQ 5 Multicolor Real-Time PCR detection System. Total RNA was extracted with the *mir*Vana™ miRNA Isolation Kit (Ambion, Cat# AM1560), following manufacturer's instructions. After verifying concentration and integrity, 1 µg of total RNA was utilized to perform a RNase free DNase digestion and for first strand cDNA synthesis with random primers (N_9_) (Superscript II reverse transcriptase, Invitrogen Cat# 18064-014). PCR with primers specific for wheat rRNA (For 5′- TGGCACATTACGTGCCCG, Rev 5′- CTACCAGCACGGCCATCG) as well as the Actin2 gene product (For 5′-GCTGGATTCTGGTGATGGTGTGAG, Rev 5′-CAATGAGAGATGGCTGGAAGAGGAC) was performed with the BIO-RAD IQ SYBR Green Supermix (BIO-RAD Cat# 170-8880S). The Actin control gene was analyzed for three replicates of cDNA from each genome (dilution factor 1∶10) and wheat-specific rDNA gene expression was quantified for two replicates of two cDNA dilutions (1∶100 and 1∶500). Each 20 µL PCR mix containing forward and reverse primers (0.1 pM each) was amplified for 35 cycles (95°C-5 min, 35 cycles of 95°C 1 min, 60°C 1 min, and 72°C 1 min, and a final elongation step of 72°C for 5 minutes). Melt curves were performed to ensure amplification of single products as well as to estimate their melting temperatures. Upon completion, PCR products were separated by 1.5% agarose gel electrophoresis and detected by ethidium bromide staining.

To analyze the levels of wheat-specific rRNA between genomes, the mean actin cDNA threshold cycle (C_t_) was used to calculate each delta C_t_ (ΔC_t_ = C_t_ wheat rRNA – mean C_t_ actin) associated with two replicates of both dilutions for each genome (wheat, wheat + 1R1R and wheat + 7R7R). In order to compare these between wheat and wheat + 1R1R, delta delta C_t_ (ΔΔC_t_ = ΔC_t_ of interest – mean ΔC_t_ 7R) were calculated for wheat and wheat + 1R1R. These values were utilized for a type 2 two-tailed T-test as well as to calculate fold variation in expression (2 ^−mean ΔΔ Ct^).

### Fluorescence *in situ* hybridization and BrUTP incorporation in preserved nuclei

Fluorescence *in situ* hybridization (FISH) was performed in root-tip sections according to [35] using the following DNA probes: pTa71, a 9 kb EcoRI fragment of the rDNA from wheat (*Triticum aestivum*), containing the 5.8S, 18S, 25S and non-transcribed spacer sequences; and total genomic DNA isolated from leaves of rye (*Secale cereale*) cv. Imperial. Probes labeled with digoxigenin were detected by an anti-digoxigenin antibody conjugated to FITC (Boehringer), and biotin-labeled probes were detected with streptavidin-Cy3 (Boehringer).

The BrUTP incorporation was performed *in vivo* in vibratome sections following the procedure already described [35]. The detection of BrUTP incorporation involved the incubation with mouse anti-BrdU (Boehringer) followed by a second incubation on a secondary fluorescent anti-mouse antibody [Alexa-568 (Molecular Probes)]. Confocal optical section stacks were collected using a Leica TCS SP confocal microscope (Leica Microsystems, Heidelberg GMbH, Germany). 3D models were made from stacks of consecutive confocal sections using Object-Image (from NIH) and final images were composed using Adobe Photoshop 5.0 (Adobe systems Inc., Mountain View, CA).

### Evaluation of rDNA expression levels through silver-staining

A comparative analysis of rDNA *loci* expression levels was performed after silver-staining on root-tip cells following the procedure indicated in [23], [27] which allowed for discrimination between nucleolar chromosomes 1B, 6B and 1R.

Silver-stained NORs (Ag-NORs) were scored per metaphase cell, and classified using as a reference the length of short arm satellite of each nucleolar chromosome. This eliminated errors due to variation in chromosome condensation between different metaphases. NORs larger than half the size of the associated satellite were classified as large; those with a length between half and a quarter as medium; and those with length of less than a quarter as small.

## Supporting Information

Figure S1 Localization and size of a wheat-specific rDNA transcribed sequence. Localization and size of a wheat-specific rDNA transcribed sequence. Localization and size (bp) of the fragment expected from the amplification of a wheat-specific rDNA transcribed sequence in the ETS (External Transcribed Spacer) of Wheat rDNA 25S-18S intergenic region (IGS, accession number X07841). ↑ - transcription initiation site, ⇑ - primers location (For +830, Rev +1132).
(7.50 MB TIF)
