# Supplementary figures and images for: Interplay of Ribosomal DNA Loci in Nucleolar Dominance: Dominant NORs Are Up-Regulated by Chromatin Dynamics in the Wheat-Rye System
Source: PLoS One. 2008 Dec 2;3(12):e3824. doi: 10.1371/journal.pone.0003824 (PMC2585015; doi:10.1371/journal.pone.0003824)

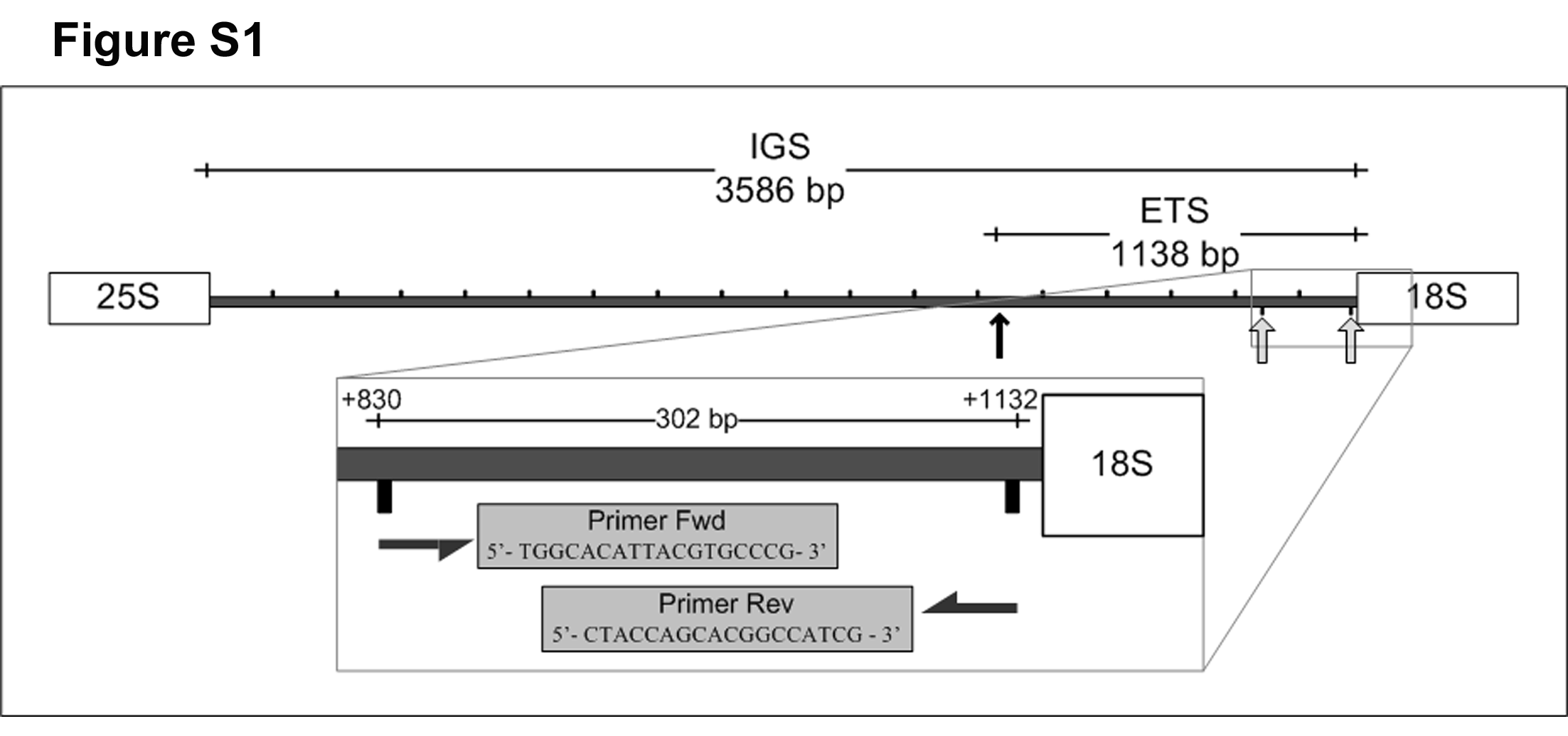

Supplement: Figure S1 — Localization and size of a wheat-specific rDNA transcribed sequence. Localization and size of a wheat-specific rDNA transcribed sequence. Localization and size (bp) of the fragment expected from the amplification of a wheat-specific rDNA transcribed sequence in the ETS (External Transcribed Spacer) of Wheat rDNA 25S-18S intergenic region (IGS, accession number X07841). ↑ - transcription initiation site, ⇑ - primers location (For +830, Rev +1132). (7.50 MB TIF) [file pone.0003824.s001.tif]
